# Doping knowledge, attitudes, and practices of Ugandan athletes’: a cross-sectional study

**DOI:** 10.1186/s13011-015-0033-2

**Published:** 2015-09-22

**Authors:** Haruna Muwonge, Robert Zavuga, Peninnah Aligawesa Kabenge

**Affiliations:** Department of Physiology, School of Biomedical Sciences, College of Health Sciences, Makerere University, P.O Box 7072, Kampala, Uganda; Department of Biomedicine, University of Bergen (Norway), Bergen, Norway; Uganda Olympic Committee (UOC), Heskethbell Road, Plot 2-10, Lugogo Sports Complex, P.O.Box 2610, Kampala, Uganda; Uganda Peoples Defense Forces (UPDF), Kampala, Uganda; Department of Sports and Recreation, Makerere University, P.O.Box 7062, Kampala, Uganda

**Keywords:** Uganda, Doping, Sport, Attitudes, Knowledge, Doping practices, Performance enhancing substances, Substance use

## Abstract

**Background:**

Despite the development of advanced drug testing systems, both deliberate and inadvertent doping in sports is increasing in elite, amateur and school sports. As a result, alternative approaches that seek to influence an athlete’s attitudes are needed to address the growing doping concerns that threaten both the health and well being of the athlete as well as the legitimacy of the sport. Therefore, the current study set out to establish the doping attitudes, knowledge and practices of professional Ugandan athletes, gathering information that may guide the design of more efficient doping prevention programs.

**Methods:**

This was a cross-sectional study of 384 professional Ugandan athletes from four contact team sports (basketball, football, handball and rugby) and two individual sports (athletics and cycling). An Interviewer administered questionnaire used contained; questions about the doping behavior, the performance enhancement attitude scale (PEAS), and doping use belief (DUB) statements.

**Results:**

Approximately 60 % of the athletes reported familiarity with information on doping and that most of this information came from fellow colleagues (41.9 %), individual or team coaches (29.7 %) or the media (15.6 %). However, nearly 80 % of these athletes could not correctly define doping. The overall mean PEAS score, a measure of doping attitudes, for all study participants was 39.8 ± 14.8. Female athletes (PEAS: 41.1 ± 15.1), athletes with a prior doping history (PEAS: 44.1 ± 15.6) and athletes from the sport of athletics (PEAS: 56.6 ± 17.4) had higher mean PEAS scores than their respective counterparts. Regarding doping behaviors/practices, 9.3 % of the study participants had been offered a doping agent at some point, although only 3.9 % of the athletes acknowledged recent use.

**Conclusions:**

The confessed use of doping agents in this study was low, which may suggest that fewer athletes use doping agents in Uganda. However, there is still an urgent need for educational anti-doping programs to address the knowledge gaps observed amongst athletes in this study. Modifying the existing Physical education curriculum for inclusion of more content about doping in sport could provide the basis for doping prevention programs amongst amateur athletes in Ugandan primary and secondary schools.

**Electronic supplementary material:**

The online version of this article (doi:10.1186/s13011-015-0033-2) contains supplementary material, which is available to authorized users.

## Background

Despite the development of advanced drug testing systems, both deliberate and inadvertent doping in sports is increasing in elite, amateur and school sports [1–4]. According to the 2013 World Anti-doping Agency (WADA) report, the number of abnormal test findings recorded by anti-doping authorities worldwide have increased by more than 20 % since 2012 [5]. One biochemical analysis of 7,289 blood samples collected globally from 2,737 track and field athletes both out of and during competition from 2001 to 2011 found a 14 % mean period prevalence of blood doping, with a range of 1 % to 48 %, depending on the nationality of the athletes [6]. In the general population, a meta-analysis of studies done in the African region for the period between 1970–2013, found a 2.4 % lifetime prevalence of anabolic-androgenic steroid use [7].

Therefore, in a bid to deter athletes from using banned performance enhancing drugs/methods, athletes have been subjected to impromptu in- and out-of-competition screening tests for these substances over time, and those athletes who test positive are given heavy punishments or fines. However, despite the rigorous testing procedures, the decades of doping scandals that have nonetheless prevailed have shown that the tests are no guarantee of a drug-free race. It is difficult to name a tour de France, an Olympic competition or even a Commonwealth competition in recent years that has gone unmarred by doping accusations. Ewen Callaway, in a Nature feature article, appropriately termed it “an endless cycle,” where anti-doping agencies try to thwart one cheating strategy while another emerges [8].

Although anti-doping control testing is obviously necessary, other programs aimed at discouraging athletes to use banned substances are sorely needed. Support for such programs was reinforced in a speech by the president of the International Olympic Committee, Thomas Bach, who remarked that “there should be a change in emphasis from fighting against drugs in sport, to protecting a clean athlete” [9]. In principle, most of these parallel programs focus on influencing athletes’ attitudes and beliefs toward the use of performance enhancing substances. In one such approach, the psychosocial approach, attitudes are considered an index of doping behavior, and a greater leniency towards doping is linked to the use of banned substances [10]. In a meta-analysis by Ntoumanis et. al on personal and psychosocial predictors of doping use in physical activity settings, a positive attitude towards doping was one of the strongest positive correlates to doping intentions and behaviors [11]. Therefore, a greater understanding of an athlete’s knowledge of, attitudes toward, and practices in doping is crucial for developing efficient prevention programs. However, despite the potency of such information in designing national anti-doping programs, there is still a paucity of data on doping in the majority of sub-Saharan countries, including Uganda. In a necessary bid to provide an impetus on which Uganda’s’ anti-doping programs could be based, this study set out to determine the knowledge, attitudes and practices of Ugandan athletes towards doping.

Several studies of a similar nature [10, 12–15] have been conducted in other parts of the world, where athletes have access to more advanced training facilities, more resources to acquire more sophisticated doping substances/methods, and a greater access to databases of knowledge on doping and its consequences. Because the issue of doping is complex, and is presumably predicted by a variety of situational and personal factors [11], results from the above studies cannot entirely be extrapolated to athletes in the Sub-Saharan region, where training facilities are poor, resources to access the most potent and least detectable doping substances/methods are lacking, and where ready access to databases with information about doping is still low.

## Methods

### Study design

This was a cross sectional study involving 384 professional Ugandan athletes from six sporting disciplines; four contact sports (basketball, football, handball, and rugby) in the major national league and two endurance sports (athletics and cycling).

The Uganda Olympic Committee (UOC) in conjunction with the Regional anti-doping agency (RADO) for the East African region lead, and coordinate the doping prevention efforts in the country.

### Participants

In the current study, we only enrolled athletes over 18 years of age who were currently playing at a professional level for a club. Participants who had retired from a sport or those who had not participated in a competitive game or competition in the past year were excluded. Of the 384 athletes who were approached to participate in the study, 360 consented and gave complete responses, resulting in a response rate of 93.75 % for the current study. The mean age of the athletes was 24 years. The majority of the interviewed athletes (Table 1) were male (60.6 %), and most had at least attained tertiary education, which is equivalent to a diploma and above, from a vocational school, college or university. Fifty nine participants (16.3 %) were rugby players, 61 (16.9 %) participants played basketball, 60 (16.6 %) were cyclists, 59 (16.3 %) participants played hand ball, 61 (16.9 %) participants were footballers, and 61 (16.9 %) participants were track and field athletes’. The study sample size was estimated using the Kish and Leslie formula [16] for cross-sectional studies to give a power of 80 %. Stratified random sampling was used in the recruitment of study participants. Independent variables for the study included the following: demographic characteristics of the participants (age, sex, marital status, level of education, and occupation) and the type of sport. The dependent variables were the following: the athletes’ knowledge of doping, doping beliefs, doping attitudes and doping practices.

**Table 1:** Sociodemographic characteristics

Age (years)	24 ± 4.8
Gender (*n* = 360)	
Male	218 (60.6 %)
Female	142 (39.4 %)
Level of education (*n* = 348)	
Primary	7 (2.0 %)
Secondary	97 (27.9 %)
Tertiary	244 (70.1 %)
Sport (*n* = 361)	
Rugby	59 (16.3 %)
Basketball	61 (16.9 %)
Cycling	60 (16.6 %)
Handball	59 (16.3 %)
Football	61 (16.9 %)
Athletics	61 (16.9 %)
Mean duration the sport was played (years)	4.49 ± 3.8

### Instruments

An interviewer-administered questionnaire was used to collect the data. The questionnaire contained questions from the *Performance Enhancement Attitude Scale* (PEAS) [17], *Doping Use Belief* (DUB) statements, questions regarding past experience and current use of doping, brief definitions of terminology (i.e., performance enhancing drugs and methods), and a cover letter explaining the purpose of the study [18].

The *Performance Enhancement Attitude Scale* (PEAS) is a measure of general doping attitudes [17, 18], and as such was used for that purpose in the current study. The doping attitude is defined as an individual's predisposition toward the use of banned performance enhancing substances and methods [18]. The PEAS consists of 17 attitude statements, which are measured on a six point Likert-type scale ranging from strongly disagree (1) to strongly agree (6). No neutral middle point is offered, and all 17 items are scored in the same direction. A range of 17–102 is possible, with a higher score indicating a more positive attitude toward doping. Previous studies that have used the tool concluded that the scale is uni-dimensional and reliable, with Cronbach’s alpha values ranging from 0.71 to 0.91 [17]. In the current study, the Cronbach’s alpha value for the PEAS was 0.8867.

*Doping Use Belief* measures (DUB) are defined as expressions of the presumed opinion regarding doping use [18], i.e., whether doping should be allowed for top and all level athletes (2 separate questions). Participants were asked to select one of the 3 responses: 'yes, without restrictions', 'yes, with restrictions' and 'absolutely not'. However, the original questions were modified to suit the current study population. The *Doping behavior/practice* was defined by two self-reported measures of doping behavior, i.e., current use of and past experience with performance enhancing substances/methods. During the interviews, reference was made to the list of banned performance substances/methods, which was contained in the survey questionnaire, but it was not shown to the study participants before or during the interviews.

### Procedure

Athletes were guaranteed complete anonymity, and written informed consent was obtained from each athlete before participating in the study. The participants were then interviewed individually, usually at the club premises or at the training grounds before or after training sessions. Prior to data collection, research assistants were trained on the use of the questionnaire, which was then pretested. Data were entered into Microsoft Excel 1997–2003 software program and thereafter exported to the Stata software (StataCorp. STATA 12.1, College Station, TX, USA) for analysis. Data are presented as the frequencies, percentages and means with standard deviations.

### Statistical analysis

We assessed the Performance Enhancement Scale (PEAS)—Seventeen six-point questions ranging from strongly disagree to strongly agree, for reliability using Cronbach’s alpha. This scale demonstrated a high coefficient of reliability (alpha = 0.8867), and therefore a relatively high internal consistency. (See Additional file 1: for a copy of the PEAS).

We summarized all continuous variables (e.g. Age and total score from PEAS) using mean and standard deviation (i.e. mean(SD)), after assessing for normality of the data. Categorical variables were summarized using frequencies and percentages. We compared total PEAS scores across the gender and doping experience using an independent sample t-test, and type of sport using one way ANOVA and Scheffe’s test as posthoc: a *P value* <0.05 was considered significant. Permission to perform the study was obtained from the Institutional review board of the School of Biomedical Sciences (MU-CHS).

## Results

### Doping Knowledge

When participants were asked whether they had received information regarding banned substances in their respective sport (Table 2), two-thirds of the athletes replied in the affirmative. Most of this information was reported to have come from fellow colleagues (41.9 %), coaches (29.7 %) and the media (15.6 %). Federation officials, health professionals and the Internet were the other sources of information on banned substances.

**Table 2:** General knowledge about doping

Received information^a^	
Yes	226 (62.9 %)
Male	145 (66.8 %)
Female	81 (57.1 %)
No	133 (37.2 %)
Male	72 (33.1 %)
Female	61 (42.9.0 %)
Source of information	
Colleagues	89 (41.9 %)
Media	33 (15.6 %)
Coach	63 (29.7 %)
Federation officials	13 (6.1 %)
Health professionals	6 (2.8 %)
Internet	5 (2.4 %)
Others	3 (1.4 %)
Knowledge confidence^b^	
Yes	135 (37.7 %)
No	223 (62.3 %)

^a^Have you received information about banned substances in your sport?

^b^Are you confident in your knowledge about banned substances in your sport?

Items that examined the athletes’ knowledge about the definition of doping were extracted from the 2009 World anti-doping code [19]. With respect to the definition of doping (Table 3), 10 % of the athletes acknowledged a deficit in knowledge. However, more than 80 % failed to give a correct answer when asked if doping involved the following actions: administration of banned substances by a doctor, refusing and tampering with doping sample collection, and whether it involved trafficking of prohibited substances by the coach. More than two-thirds of the athletes also gave false answers when asked if doping is defined as the inadvertent use of prohibited drugs by athletes or involves the presence of a prohibited substance in a doping urine sample.

**Table 3:** Knowledge about the definition of doping

Administration^a^ (*n* = 359)	
Yes	67 (18.7 %)
No	292 (81.3 %)
Announcement^b^ (*n* = 358)	
Yes	13 (3.6 %)
No	345 (96.4 %)
High altitude training^c^ (*n* = 359)	
Yes	59 (16.4 %)
No	300 (83.6 %)
Prohibited drugs^d^ (*n* = 358)	
Yes	139 (38.8 %)
No	219 (61.2 %)
Nutritional supplements^e^ (*n* = 358)	
Yes	58 (16.2 %)
No	300 (83.8 %)
Substance in urine sample^f^ (*n* = 358)	
Yes	88 (24.6 %)
No	270 (75.4 %)
Refusing sample collection^g^ (*n* = 358)	
Yes	62 (17.3 %)
No	296 (82.7 %)
Tampering^h^ (*n* = 358)	
Yes	46 (12.8 %)
No	312 (87.2 %)
Trafficking^i^ (*n* = 358)	
Yes	35 (9.8 %)
No	323 (90.2 %)
Don’t know (*n* = 358)	
Yes	35 (9.8 %)
No	323 (90.2 %)

^a^Administration of banned substances by a doctor

^b^Announcement of special financial rewards for moral enhancement

^c^Enhancing performance with high altitude training

^d^Inadvertent use of prohibited drugs by athletes

^e^Power enhancement using special nutritional supplements

^f^Presence of prohibited substance in doping urine sample

^g^Refusing to undergo doping sample collection

^h^Tampering with doping sample collection

^i^Trafficking in prohibited substances by coach

Athletes displayed varying levels of awareness regarding the different key thematic areas in doping (Table 4). Generally, the majority of the interviewed athletes had an idea about the different components of doping prevention, such as the prohibited list, anti-doping testing procedures and anti-doping rule violations. Of the six anti-doping prevention themes considered, the subject of therapeutic use exemptions regarding doping stood out as the subject that was the least known; more than half of the participants had absolutely no knowledge on that subject.

**Table 4:** Do you have knowledge about the following aspects of doping?

Item	To a large extent	To some extent	No
The prohibited list (*n* = 360)	72 (20 %)	173 (48.1 %)	115 (31.9 %)
Therapeutic^a^ (*n* = 358)	63 (17.6 %)	112 (31.3 %)	183 (51.1 %)
Procedures^b^ (*n* = 357)	75 (21.0 %)	148 (41.5 %)	134 (37.5 %)
Health risks^c^ (*n* = 357)	133 (37.2 %)	156 (43.7 %)	68 (19.1 %)
Rule violations^d^ (*n* = 357)	85 (23.8 %)	158 (44.3 %)	114 (31.9 %)
Sanctions^e^ (*n* = 357)	92 (25.8 %)	116 (32.5 %)	149 (41.7 %)

^a^Therapeutic use exemptions

^b^Procedures for anti-doping testing

^c^Health risks related to doping

^d^Anti-doping rule violations

^e^Sanctions on anti-doping rule violations

### Doping attitudes

The overall mean PEAS score for all study participants was 39.8 ± 14.8. The PEAS score of male athletes (Table 5) did not differ significantly from female athletes in the current study. Using one-way ANOVA, there were significant differences between groups compared to within groups (see Table 5). PostHoc analysis using Scheffe’s test showed significant mean differences between the PEAS of athletes and all the other sporting groups.

**Table 5:** Performance Enhancement Attitude Scale (PEAS) score

Item	Mean (SD) PEAS	*P*-value
Overall	39.8 ± 14.8	
Gender		0.184^a^
Male	39.0 ± 14.6	
Female	41.1 ± 15.1	
Sport		0.001^b^
Rugby	34.0 ± 11.4	
Basketball	37.7 ± 8.5	
Cycling	38.9 ± 14.3	
Handball	36.5 ± 11.1	
Football	34.8 ± 11.7	
Athletics	56.6 ± 17.4	
Doping experience		0.0985^a^
Yes	44.1 ± 15.6	
No	39.4 ± 14.7	

^a^student’s t-test with df = 358

^b^F-test from ANOVA, df (5.0, 5.4)

### Doping beliefs

Concerning athlete doping beliefs (Table 6), the majority of the participants did not believe that athletes at any level should be allowed to use performance-enhancing drugs/methods.

**Table 6:** Modified Doping Use Belief (DUB)

Believe^a^ (*n* = 360)	
Yes, without restrictions	13 (3.6 %)
Yes, but with restrictions	50 (13.9 %)
Absolutely not	297 (82.5 %)
Allowed^b^ (*n* = 359	
Yes, without restrictions	11 (3.1 %)
Yes, but with restrictions	57 15.9 %)
Absolutely not	291 (81.0 %)

^a^Do you believe that performance-enhancing drugs/methods should be allowed for top-level athletes?

^b^Do you believe that performance-enhancing drugs/methods should be allowed for all athletes?

### Doping practices

Nine point three percent of the participants had ever been offered a doping agent/methods by their colleagues, a member of the coaching staff or a member of the family (Table 7). Additionally, precisely 3.9 % of the interviewed athletes acknowledged having ever used a banned performance-enhancing drug/method in their life, with 3.3 % of them admitting to recent use. However, slightly more than 10 % of the athletes declined to give a response when asked if they had ever used the banned performance-enhancing drugs/methods. Amongst athletes who admitted to past use of doping substances, majority were either cyclists (23.3 %) or rugby players (23.3 %) (Table 8). Concerning the extent of doping in the sports community, 13.1 % of the athletes interviewed were certain that they knew someone in the sports community (Table 7) who has used doping substances, and 23 % believed they knew someone who has used doping substances or methods but were not certain.

**Table 7:** Doping practices

Offered^a^ (*n* = 346 %)	
Yes	32 (9.3 %)
No	314 (90.7 %)
Personal experience^b^ (*n* = 360)	
Yes	14 (3.9 %)
Yes, but only for treating a medical condition	16 (4.4 %)
No	289 (80.3 %)
I do not wish to answer	41 (11.4 %)
Current use^c^ (*n* = 360)	
Yes	12 (3.3 %)
Yes, but only for treating a medical condition	12 (3.3 %)
No	296 (82.2 %)
I do not wish to answer	40 (11.1 %)
Knowledge^d^ (*n* = 360)	
Yes, certainly	47 (13.1 %)
I believe so, but am not sure	82 (22.8 %)
No	231 (64.1 %)

^a^Have you been offered doping agents/methods?

^b^Have you ever had personal experience with banned performance-enhancing drugs and/or methods?

^c^Do you currently use banned performance-enhancing drugs?

^d^Do you know people in your sports community who have used doping?

**Table 8:** Personal experience with doping substances

Sport	Yes	No
Rugby	7 (23.3 %)	52 (15.8 %)
Basketball	3 (10 %)	58 (17.6 %)
Cycling	7 (23.3 %)	53 (16.1 %)
Handball	4 (13.3 %)	55 (16.7 %)
Soccer	4 (13.3 %)	57 (17.3 %)
Athletics	5 (16.7 %)	55 (16.7 %)

## Discussion

The present cross-sectional survey was performed to assess the knowledge, beliefs, attitudes and practices of Ugandan professional athletes toward doping. In this study, standard doping attitudes and behavior survey tools were used to collect the data. Overall, majority of athletes surveyed were familiar with information regarding banned substances in sports, although fewer than 20 % could correctly define various acts of doping in sports as specified by the World anti-doping agency. Also, cyclists and athletes from the sport of athletics expressed a greater permissiveness to doping, compared to athletes from other sports. However, the overall reported use of doping substances/methods amongst the athletes in the current study was relatively low.

Generally, it is believed that an individual’s state of knowledge is normally influenced by their education status. In the current study, majority of the athletes had at least completed secondary school. As such, we postulate that this educational background could have contributed to the fact that most of them acknowledged that they were relatively well informed about banned substances/methods in sports. Nevertheless, these findings complement those from similar studies involving other athletes from Europe, North America or the United Kingdom. For instance, Erdman et al. reported that 76.7 % of 582 high-performance Canadian athletes were aware of the anti-doping regulations [20], whereas Waddington et al. [21] found a 68 % familiarity of the UK sport guidelines on banned drug use among 706 members of the English Professional Football Association (PFA). These observations could have potential implications in the implementation of doping awareness programs, whereby academic institutions could serve as one of the avenues for dissemination of anti-doping messages.

As observed elsewhere [10, 12, 20, 22–24], the major sources for doping information in this study were colleagues and coaches. For example, in a study involving British junior team athletes, Nieper [23] observed that the coaches provided most of the information regarding doping in sports, whereas Erdman et al. noted that family/friends and team mates were the most common sources of information on the use of PES in a group of 582 high-performance Canadian athletes [20]. In contrast to what was observed in the current study, Somervile et al. [24] reported that the team doctor was the most popular source of information on PES during a survey of 196 British Olympic-level athletes. Due to limited resources, the coverage of anti-doping educational programs in Uganda is still low. The additional fact that the existing curriculum and syllabus for Physical Education currently implemented in primary and secondary schools across the country, being limited in sports doping content has not helped either. Such circumstances make other alternative informal sources of information, such as the coaches, fellow athletes, and the media, very important. Therefore it can be implied from this data that doping prevention programs designed to target this group of individuals could significantly create a huge impact on athlete doping knowledge and attitudes.

Although most athletes’ in the current study had acknowledged a modest familiarity to anti-doping information, only a handful could correctly define doping as stipulated by the WADA. This finding raises important questions regarding the content of the existing athlete doping awareness packages and the coverage/reach of such prevention programs. More so, it could potentially be a limitation of the existing doping prevention programs, one that can be remedied through appropriate educational programs, as suggested by Morente-Sanchez and colleagues [10]. Additionally, it is still worth mentioning that the insufficient media coverage of doping-related themes and a lack of realization that doping might be a serious concern in Ugandan sport may perhaps explain the overwhelming lack of knowledge in certain aspects of doping observed among athletes in the current study.

As used elsewhere [17, 25], the performance enhancement attitude scale (PEAS) was the chosen measure of general doping attitudes in the current study. Because attitudes could be considered as predictors of doping behavior [26], knowledge and alteration of an athletes’ attitude towards the use of banned performance-enhancing substances is one of the most important goals in the worldwide effort to prevent sports doping [14]. In this regard, we examined for an association between the athletes’ doping attitudes with gender, the nature of sport played, and the current use of doping agents/methods.

There were no significant gender specific differences in the PEAS scores between male and female athletes in the current study (*p* = 0.184). This finding is in agreement with data from previous studies, which had established male athletes to be more permissive to the use of doping substances/methods than their female counterparts [13, 27]. Additionally, cyclists and track and field athletes’ in the present study displayed a significantly greater permissiveness to the use of doping substances/methods (*p* = 0.036 & *p* <0.001 respectively) than other athletes from rugby, basketball, football and handball. What is peculiar about the nature of this association is that both cycling and track and field athletics are categorized as individual sports, whereas basketball, football, rugby and handball are team sports. A study involving 750 elite Greek athletes, Lazaras et. al also observed that the use of doping substances was more common (*p* <0.005) amongst athletes in individual sports (14.4 %), than in team sports (7.4 %) [28]. In a review about elite athlete doping attitudes, belief, and knowledge, Morente-Sanchez et. al suggests that the above differences observed in doping attitudes between sports could be related to either the independence of different sports federations, or the differences in the number and quality of doping controls in each sport, which usually differ substantially (eg. More controls in cycling Vs Football) [10]. In consideration of the relationship between admitted use of doping agents and doping attitudes, this study did not find any association (*p* = 0.098), despite prior studies finding a significant and direct relationship [11, 19].

Doping prevalence is not easy to measure, and previous epidemiologic studies have reported wide variance [29]. Nonetheless, depending on the definition, the proportion of athletes using doping agents as determined by prior questionnaire surveys, ranged between 1.3 and 39.2 % [29], with a mean of 14 %, when the athlete’ biological passport was used to ascertain the prevalence of blood doping for samples collected in the period between 2001–2011 [6]. The reported use of doping substances/methods amongst athletes in the current study was relatively low (3.3 %), although the general implied use after participants were asked if they knew a person who was currently using doping substances/methods was relatively high (13.1 %). One possible explanation for this disparity could be that people tend to considerably overestimate the proportion of people who engage in a behavior that they also engage in, a special type of bias that is termed the “false consensus” effect [30]. However, it is still reasonable to assume that only 3.3 % of the study participants were in fact engaged in doping in the current study, as a similar proportion (3.9 %) of high school athletes in the Gauteng province of South Africa had admitted to using doping substances/methods [12]. This figure is also close to that computed from a meta-analysis of studies done in the African region during the period between 1970–2013, where the lifetime prevalence of anabolic-androgenic steroid was 2.4 % [7].

To the best of our knowledge, this is the first report on the assessment of the knowledge, attitudes and practices of Ugandan athletes towards doping. Although these results cannot be directly generalized to other sub-Saharan countries, they could be extrapolated to regions or countries with inadequately resourced anti-doping programs, many of which are found in Africa. Therefore, these data will help in formulating focused, informed, and gender-based anti-doping awareness interventions aimed at utilizing the meager resources for pursuing evidence-based anti-doping activities. Because the lack of appropriate anti-doping knowledge may be partially attributed to the inadequate sports education curriculum in schools, this report will provide a platform for initiating conversations and eliciting suggestions on the revision of the sports education curriculum in Uganda.

As in other standard questionnaire surveys, this study relied on the respondents to provide honest responses to the survey questions. The participants’ responses, although invaluable, are susceptible to responder bias, particularly in situations in which the study participants give socially desirable responses when confronted with questions about indulgence in socially unacceptable habits, such as doping. Therefore, future efforts should consider using more reliable survey methods for estimating doping prevalence, like biochemical testing. Another study limitation was the lack of local data on doping in Uganda, which made the selection of survey tools and prior determination of the sample size equally difficult. Therefore, standardized doping survey tools, such as the PEAS and DUB questions, which had been validated and used to assess doping attitudes and behavior in other regions, were employed in the current survey.

## Conclusions

The confessed use of doping agents in this study was low, which may suggest that fewer athletes use doping agents in Uganda. However, there is still an urgent need for anti-doping educational programs to address the knowledge gaps observed amongst athletes in this study. Such programs should not only target athletes but should also involve coaches, doctors and members of the athletes family, whose relationship with the athletes may either act to encourage or minimize doping behavior. Furthermore, additional advocacy should be made to ensure the strict implementation of a comprehensive sports education curriculum in schools, where information about doping can be emphasized at an early stage. In turn, this could form the basis for doping prevention programs amongst amateur athletes.

## Additional file

Additional file 1:
**Performance Enhancement Attitude Scale** **(PEAS)**. (DOC 48 kb)

## Abbreviations

PEA: Performance enhancement attitudes
PEAS: Performance enhancement attitudes score
RADO: Regional Anti-doping organization
UOC: Uganda Olympic committee
DUB: Doping use beliefs
WADA: World Anti-doping agency
MoES: Ministry of Education and Sports
NCS: National Council of Sports
IOC: International Olympic committee
PES: Performance enhancement substances

## References

[1] BBC Sport: Anti-doping testing: abnormal results rise by 20 % in 2013. In: BBC Sport. Edited by BBC; 2014. http://www.bbc.com/sport/0/28194582.

[2] de Hon O, Kuipers H, van Bottenburg M (2015). Prevalence of doping Use in elite sports: a review of numbers and methods. Sports Med.

[3] Pielke R (2015). Gather data to reveal true extent of doping in sport. Nature: Int Weekly J Sci.

[4] Gibson O (2013). Drugs in sport: Wada says doping and organised crime 'too big to manage'. theguardian.

[5] World Anti-doping Agency (WADA), World anti-doping Agency (2013). Anti-Doping Testing figures. 2013 Anti-Doping Testing Figures Report.

[6] Sottas PE, Robinson N, Fischetto G, Dolle G, Alonso JM, Saugy M (2011). Prevalence of blood doping in samples collected from elite track and field athletes. Clin Chem.

[7] Sagoe D, Molde H, Andreassen CS, Torsheim T, Pallesen S (2014). The global epidemiology of anabolic-androgenic steroid use: a meta-analysis and meta-regression analysis. Ann Epidemiol.

[8] Ewen C (2011). Sports doping: racing just to keep up. Nature: News Feature.

[9] World Anti-doping Agency (WADA) (2014). Annual Report.

[10] Morente-Sanchez J, Zabala M (2013). Doping in sport: a review of elite athletes' attitudes, beliefs, and knowledge. Sports Med.

[11] Ntoumanis N, Ng JY, Barkoukis V, Backhouse S (2014). Personal and psychosocial predictors of doping Use in physical activity settings: a meta-analysis. Sports Med.

[12] Nolte BS K, Fletcher L, Krüger PE (2014). Doping in sport: attitudes, beliefs and knowledge of competitive high-school athletes in Gauteng province. South African J Sports Med.

[13] Alaranta A, Alaranta H, Holmila J, Palmu P, Pietila K, Helenius I (2006). Self-reported attitudes of elite athletes towards doping: differences between type of sport. Int J Sports Med.

[14] Brand R, Wolff W, Thieme D (2014). Using response-time latencies to measure athletes' doping attitudes: the brief implicit attitude test identifies substance abuse in bodybuilders. Subst Abuse Treat Prev Policy.

[15] Peretti-Watel P, Pruvost J, Guagliardo V, Guibbert L, Verger P, Obadia Y (2005). Attitudes à l’égard du dopage parmi les jeunes sportifs de la région PACA. Sci Sports.

[16] Kish L (1965). Survey Sampling.

[17] Petróczi A, Aidman E (2009). Measuring explicit attitude toward doping: review of the psychometric properties of the performance enhancement attitude scale. Psychol Sport Exercise.

[18] Petróczi A (2007). Attitudes and doping: a structural equation analysis of the relationship between athletes' attitudes, sport orientation and doping behaviour. Substance Abuse Treatment, Prevention, and Policy C7 - 34.

[19] World anti-doping Agency (2009). World Anti-Doping Code. Doping Control: Definition of doping.

[20] Erdman KA, Fung TS, Doyle-Baker PK, Verhoef MJ, Reimer RA (2007). Dietary supplementation of high-performance Canadian athletes by age and gender. Clin J Sport Med.

[21] Waddington I, Malcolm D, Roderick M, Naik R (2005). Drug use in english professional football. Br J Sports Med.

[22] Sajber D, Rodek J, Escalante Y, Olujic D, Sekulic D (2013). Sport nutrition and doping factors in swimming; parallel analysis among athletes and coaches. Coll Antropol.

[23] Nieper A (2005). Nutritional supplement practices in UK junior national track and field athletes. Br J Sports Med.

[24] Somerville SJ, Lewis M, Kuipers H (2005). Accidental breaches of the doping regulations in sport: is there a need to improve the education of sportspeople?. Br J Sports Med.

[25] Manouchehri J, Farshad T, Farideh Ashraf G (2013). Variance analysis: doping attitude, doping behavior and sport orientation in elite martial artists european. J Exp Biol.

[26] Kraus SJ (1995). Attitudes and the prediction of behavior: a meta-analysis of the empirical literature. Personal Soc Psychol Bull.

[27] Sas-Nowosielski K, Swiatkowska L (2008). Goal orientations and attitudes toward doping. Int J Sports Med.

[28] Lazuras L, Barkoukis V, Rodafinos A, Tzorbatzoudis H (2010). Predictors of doping intentions in elite-level athletes: a social cognition approach. J Sport Exerc Psychol.

[29] Lentillon-Kaestner V, Ohl F (2011). Can we measure accurately the prevalence of doping?. Scand J Med Sci Sports.

[30] Petroczi A, Mazanov J, Nepusz T, Backhouse SH, Naughton DP (2008). Comfort in big numbers: does over-estimation of doping prevalence in others indicate self-involvement?. J Occup Med Toxicol.

