# Supplementary material for: Doping knowledge, attitudes, and practices of Ugandan athletes’: a cross-sectional study
Source: Subst Abuse Treat Prev Policy. 2015 Sep 22;10:37. doi: 10.1186/s13011-015-0033-2 (PMC4579610; doi:10.1186/s13011-015-0033-2)
Supplement: Additional file 1: — Performance Enhancement Attitude Scale (PEAS). (DOC 48 kb) [file 13011_2015_33_MOESM1_ESM.docx]

**Performance Enhancement Attitude Scale (PEAS)**

Below are statements showing what many people think and feel about sport and performance enhancing drugs. How strongly do you agree or disagree with the following statements?

|  | **Strongly disagree** | **Disagree** | **Slightly disagree** | **Slightly agree** | **Agree** | **Strongly agree** |
| --- | --- | --- | --- | --- | --- | --- |
| 1. Doping is necessary to be competitive. |  |  |  |  |  |  |
| 2. Doping is not cheating since everyone does it. |  |  |  |  |  |  |
| 3. Athletes often lose time due to injuries and drugs can help to make up the lost time. |  |  |  |  |  |  |
| 4. Only the quality of performance should matter, not the way athletes achieve it. |  |  |  |  |  |  |
| 5. Athletes in my sport are pressured to take performance-enhancing drugs. |  |  |  |  |  |  |
| 6. Athletes, who take recreational drugs, use them because they help them in sport situations. |  |  |  |  |  |  |
| 7. Athletes should not feel guilty about breaking the rules and taking performance-enhancing drugs. |  |  |  |  |  |  |
| 8. The risks related to doping are exaggerated. |  |  |  |  |  |  |
| 9. Athletes have no alternative career choices, but sport. |  |  |  |  |  |  |
| 10. Recreational drugs give the motivation to train and compete at the highest level. |  |  |  |  |  |  |
| 11. Doping is an unavoidable part of the competitive sport. |  |  |  |  |  |  |
| 12. Recreational drugs help to overcome boredom during training. |  |  |  |  |  |  |
| 13. There is no difference between drugs, and speedy swimsuits that are all used to enhance performance. |  |  |  |  |  |  |
| 14. Media should talk less about doping. |  |  |  |  |  |  |
| 15. The media blows the doping issue out of proportion. |  |  |  |  |  |  |
| 16. Health problems related to rigorous training and injuries are just as bad as from doping. |  |  |  |  |  |  |
| 17. Legalising performance enhancements would be beneficial for sports. |  |  |  |  |  |  |
